# Prevalence and risk factors of latent tuberculosis among Korean healthcare workers using whole-blood interferon-γ release assay

**DOI:** 10.1038/s41598-018-28430-w

**Published:** 2018-07-04

**Authors:** Jeong Hwa Yeon, Hye Seong, Ho Hur, Yoonseon Park, Young Ah Kim, Yoon Soo Park, Chang Hoon Han, Sun Min Lee, Jeong Hun Seo, Jung Gu Kang

**Affiliations:** 1 Infection Control Unit, National Health Insurance Service Ilsan Hospital, Goyang, Republic of Korea; 2 Department of Surgery, National Health Insurance Service Ilsan Hospital, Goyang, Republic of Korea; 3 Department of Internal Medicine, National Health Insurance Service Ilsan Hospital, Goyang, Republic of Korea; 4 Department of Laboratory Medicine, National Health Insurance Service Ilsan Hospital, Goyang, Republic of Korea; 5 Department of Internal Medicine, Yonsei University College of Medicine, Seoul, Republic of Korea

## Abstract

Because healthcare workers (HCWs) are at high risk for tuberculosis (TB) infection, it is essential to research the prevalence of latent TB infection (LTBI) and to implement health interventions including early treatment of LTBI and TB infection control measures. The purpose of the study was to determine the prevalence and risk factors for LTBI using interferon-γ release assay (IGRA) among HCWs in South Korea. The cross-sectional study was carried in the National Health Insurance Service Ilsan Hospital, which is a 740-bed general hospital, South Korea. HCWs who participated in this survey were required to complete a questionnaire and IGRA was performed. Of the 1,655 HCWs, 271 results were positive and the prevalence of LTBI was 16% (95% CI; 15–18%). In the multivariate analysis, age (OR; 2.201, 95% CI; 1.911–2.536, P < 0.001), male sex (OR; 1.523, 95% CI; 1.133–2.046, P = 0.005), contact active TB patients (OR; 1.461, 95% CI; 1.061–2.010, P = 0.02) and diabetes (OR; 2.837, 95% CI; 1.001–8.044, P = 0.05) were significant risk factors for LTBI. LTBI among HCWs in Korea, although prevalent, might not exceed the background level of the general population. Because contact with active TB patients has been identified as a risk factor for LTBI, more effective TB infection control measures are essential in healthcare facilities and congregate settings.

## Introduction

Despite the worldwide effort to control tuberculosis (TB), it remains a significant global health problem. According to the Global TB report 2016 by World Health Organization, TB was one of the top 10 causes of death worldwide^1^. In 2015, 10.4 million people developed TB and 1.8 million, including 0.4 million individuals with human immunodeficiency virus (HIV) infection, died from the disease^1^. Latent TB infection (LTBI) is a state in which a persistent immune response occurs to *Mycobacterium tuberculosis* without any clinical manifestations of active TB disease. Although the majority of persons with LTBI have no signs or symptoms of TB, they are at risk for developing active TB disease. Prevention of new *M*. *tuberculosis* infections and progression of LTBI to active TB disease is critical to reduce the burden of this disease.

Healthcare workers (HCWs) are at high risk for TB infection and some TB develops via in-hospital infection^2^. Because HCWs working in TB-related departments are at increased risk of developing TB^3^, it is essential to research the prevalence of LTBI in HCWs and to implement health interventions including early treatment of LTBI^4,5^ and TB infection control measures.

In South Korea, children undergo mandatory Bacille Calmette-Guérin (BCG) vaccination at birth^6^. For the diagnosis of LTBI, whole-blood interferon (IFN)-γ release assay (IGRA) is a better tool than the tuberculin skin test (TST), as TST has some limitations including the tendency of easily being affected by prior BCG vaccination^7^.

The purpose of the current study was to determine the prevalence and risk factors for LTBI using IGRA among HCWs in South Korea.

## Materials and Methods

### Subjects and Settings

This cross-sectional study was carried out during an 18-month period, from July 2015 to December 2016, in the National Health Insurance Service Ilsan Hospital, which is a 740-bed general hospital in Goyang, South Korea. LTBI screening was performed to prevent nosocomial transmission according to The Korean Tuberculosis Prevention Act. Our subjects included all HCWs, including nurses, doctors, laboratory technicians, housekeeping staff, auxiliaries, administration staff, and pharmacists. We excluded HCWs with a history of tuberculosis.

### Tools of the Study

#### Questionnaire

All HCWs who participated in this survey were required to complete a questionnaire. The questionnaire consisted of items related to age, sex, profession, working duration, current workplace, history of BCG vaccination, history of active TB, and medical history, including smoking, diabetes, autoimmune disease, malignancy. Additional information obtained included history of employment in a TB-related department defined as the departments where HCWs with high possibility of contact with patients with respiratory tuberculosis are working according to Korea Centers for Disease Control and Prevention guidelines for managing tuberculosis of medical institutions; hospital ward or outpatient unit of pulmonology; hospital ward or outpatient unit of infectious diseases; bronchoscopy room; Mycobacterium tuberculosis laboratory; pulmonary function test room, intensive care unit; emergency department; pulmonology and allergy clinic of pediatrics; x-ray room of chest. Moreover, participating subjects provided their contact history with active TB, both within and outside of the hospital setting.

#### Interferon-γ release assay

QuantiFERON^®^TB Gold In-Tube (QFT-GIT; Qiagen, Germantown, USA) was performed and results of the assay were interpreted according to the manufacturer’s instructions. For each case, three heparinized blood collection tubes were used. The first tube contained heparin alone (Nil tube, negative control), the second tube contained the T cell mitogen, phytohemagglutinin (mitogen tube, positive control), and the third tube contained three tuberculosis-specific antigens: ESAT-6, CFP-10, and TB7.7 (TB antigen tube). One-milliliter aliquots of blood were drawn directly into each tube and incubated at 37 °C for 24 hours, and then centrifuged and stored at 4 °C. The result of the IFN-γ assay was determined by measuring the IFN-γ in the TB antigen minus that in the negative control tube. When the level of TB antigen-Nil was >0.35 IU/mL and >25% of the Nil tube, results was considered as positive. If the mitogen-Nil was <0.5 IU/ml or the Nil was >8 IU/ml, results was considered as indeterminate. If the TB antigen was <0.35 IU/ml and mitogen control was ≥0.5 IU/ml, the test was considered negative.

#### Statistical analysis

Data were coded and statistically analyzed using the computerized software statistical package SPSS® version 23 (IBM®, Armonk, NY, USA). Dichotomous or categorical variables were compared using the χ2 test or the Fisher exact test, as appropriate. Logistic regression was performed to detect the main independent predictors for IGRA results. Variables with a P value of <0.1 in the univariate analysis were entered into a multivariate analysis using a backward elimination method.

#### Ethics

The study design was reviewed and approved by the Institutional Review Board of the National Health Insurance Service Ilsan Hospital, Gyeonggi-do, Korea with waived written informed consent (NHIMC 2017-01-013). All methods in this study were performed in accordance with the Declaration of Helsinki.

### Data availability

The datasets generated during the current study are available from the corresponding author on reasonable request.

## Results

### Characteristics of subjects

Of a total of 1,786 HCWs, 1720 (96%) completed the questionnaire and were included in the study. Of the 1,720 included HCWs, 65 (4%) were excluded from analysis because 63 had a history of active tuberculosis and two had indeterminate results with IGRA. Among 1,655 HCWs analyzed, 423 (26%) were male and 1,232 (74%) were female. The median age of the 1,655 HCWs was 33 years (interquartile range, 26 y to 43 y). Regarding profession, 158 (10%) were doctors, 777 (47%) were nurses, 210 (13%) were technicians, 331 (20%) were housekeeping staffs/auxiliaries, 155 (9%) administration staffs, and 24 (2%) were pharmacists. The median duration of employment was 51 months (interquartile range, 15 to 153), 1,081 (65%) had been vaccinated with BCG, 200 (12%) were smokers, 89 (5%) had a family history of TB, and 478 (29%) had contact with TB patients.

### Prevalence and risk factors of latent tuberculosis among healthcare workers

Overall, of the 1,655 HCWs, 271 results were positive on IGRA and the prevalence of latent TB diagnosed using IGRA was 16% (95% CI; 15–18%). For the risk factors of LTBI, male sex (P < 0.001), age group (P < 0.001), profession (P < 0.001), working duration (P < 0.001), smoking (P < 0.001), diabetes (P < 0.001), and occupational exposure to risk area (P = 0.029) were found to be statically significant in the univariate analysis (Table 1). In the multivariate analysis, age group (OR; 2.201, 95% CI; 1.911–2.536, P < 0.001), male sex (OR; 1.523, 95% CI; 1.133–2.046, P = 0.005), contact active TB patients (OR; 1.461, 95% CI; 1.061–2.010, P = 0.02) and diabetes (OR; 2.837, 95% CI; 1.001–8.044, P = 0.05) were significant risk factors for LTBI (Table 2).

**Table 1:** Risk factors for latent tuberculosis detected via IGRA among healthcare workers: univariate analysis.

Variable	No. (%)		P-value
	IGRA positive (n = 271)	IGRA negative (n = 1384)	
**Sex**			
Male	101 (24)	322 (76)	<0.001
Female	170 (14)	1062 (86)	
**Age group**, **y**			
≤29	35 (6)	587 (94)	<0.001
30–39	69 (15)	403 (85)	
40–49	101 (25)	297 (75)	
≥50	66 (41)	97 (60)	
**Professions**			
Physician	38 (24)	120 (76)	<0.001
Nurse	94 (12)	683 (88)	
Technician	41 (20)	169 (80)	
Housekeeping staff/Auxiliary	73 (22)	258 (78)	
Administration staff	24 (15)	131 (85)	
Pharmacist	1 (4)	23 (96)	
**Work duration**, **yr**			
≤1	46 (13)	318 (87)	<0.001
>1 to ≤5	65 (12)	468 (88)	
>5 to ≤10	31 (13)	205 (87)	
>10	129 (25)	393 (75)	
**BCG vaccination**			
No	43 (17)	210 (83)	0.824
Yes	179 (17)	902 (83)	
Unknown	49 (15)	272 (85)	
**Smoking**			
No	219 (15)	1236 (85)	<0.001
Yes	52 (26)	148 (74)	
**Diabetes**			
No	263 (16)	1376 (84)	<0.001
Yes	4	8 (50)	
**Autoimmune disease**			
No	269 (16)	1375 (84)	0.871
Yes	2 (18)	9 (82)	
**Malignancy**			
No	268 (16)	1377 (84)	0.243
Yes	3 (30)	7 (70)	
**Contact active tuberculosis patients**			
No	181 (15)	996 (85)	0.086
Yes	90 (19)	388 (81)	
**Work in risk area**			
No	189 (15)	1052 (85)	0.029
Yes	82 (20)	332 (80)	

IGRA; interferon-γ release assay.

**Table 2:** Risk Factors for Latent Tuberculosis among Health care workers: Multivariate Analysis.

Risk factor	OR (95% CI)	*P* value
Age group	2.201 (1.911–2.536)	<0.001
Male sex	1.523 (1.133–2.046)	0.005
Diabetes	2.837 (1.001–8.044)	0.05
Contact active tuberculosis patients	1.461 (1.061–2.010)	0.02
Work in risk area	1.355 (0.979–1.876)	0.067

OR, odds ratio; CI, confidence interval.

## Discussion

This study determined that the prevalence and risk factors of LTBI in HCWs by using IGRA.

In this study, we determined 16% of LTBI prevalence, which was similar to that of 17.2% in an earlier, multicenter study in South Korea^8^. Although another study reported higher prevalence of LTBI in South Korea (23.2%), a larger proportion of individuals with high-risk of exposure of another study could contribute to the difference in prevalence^9^.

In South Korea, prevalence of LTBI is variable. According to the seventh Korean National Health and Nutrition Examination Survey, 2016, the prevalence of LTBI was 33.2% (95% CI: 30.9–35.6). The prevalence was 6.5% in the age group 10–19, 10.9% in 20–29, 36.4% in 30–39, 46.1% in 40–49, 48.7% in 50–59, 45.0% in 60–64 although it is difficult to compare the prevalence rate with the present study because TST was used^10^. According to the Korea Centers for Disease Control and Prevention, the prevalence of LTBI in South Korea using the IGRA test ranged from 2.1% to 34%: 2.1% among first grade high school students (mean age 15.3), 2.9% among military conscripts, 15.2% among kindergarden teachers, 17.5% among HCWs (mean age 37.9), 19.3% among nursery workers (mean age 40.6), 28.5% among workers at social welfare facilities, and 34.0% among prisoners (mean age 44.2). The difference in the prevalence rate was due to the age difference in the subject group, and there was no difference of age-specific prevalence. There was no difference according to occupation^11^.

Various risk factors, such as age^8,12^, history of close contact with a person with active TB^12–14^, work in high risk areas^15^, and employment duration as HCWs^13^, have been reported worldwide. In this study, we found several risk factors affecting the prevalence of LTBI by using IGRA. Increasing age, male sex, and contact with active TB patients were independently associated with LTBI. Despite the strict application of airborne precautions, contact with active TB patients has been identified as a risk factor for LTBI. This finding suggests that TB infection control measures in the hospital are still insufficient. To ensure that effective TB infection control measures are in place, the relative risk of TB in HCWs compared with the general population must be monitored.

To the best of our knowledge, this study was the largest, single center study to reveal the prevalence of LTBI and its related risk factors among HCWs in South Korea. IGRA is a superior tool over TST, especially in South Korea where BCG vaccination is mandatory^6,8,9,16^. Moreover, the vaccine policy until 1997, which stated that TST non-responders must be re-vaccinated with BCG at 12 years of age, may increase false positive rates of TST in South Korea^17^. For that reason, we used IGRA to determine the prevalence of LTBI in the study.

South Korea has high TB burden among high-income countries. The incidence of tuberculosis is highest from among the 34-member countries of the Organization for Economic Co-operation and Development (OECD). The incidence was 80/100,000 population in 2015, which was three times higher than that in Portugal, which reported the second highest incidence^1^. Reasons for the high burden of TB in South Korea include 1) high prevalence of LTBI in the elderly population; 2) inadequate patient management. In South Korea, LTBI should be detected for high risk HCWs by Tuberculosis Prevention Act since Aug 2016^18^.

The global prevalence of LTBI was estimated at 23.0% in 2014. The South-East Asia Region had the highest prevalence of 30.8%^19^. The current lifetime risk of progression from LTBI to active TB disease is 5–15%^20^. Given the importance of LTBI in reducing the burden of disease and death caused by TB, detection and treatment of LTBI is critical^1^.

Because HCWs are at greater risk of TB infection and disease, and nosocomial outbreaks of multidrug-resistant TB and extensively drug-resistant TB have been documented, a comprehensive set of infection control measures such as administrative, environmental and personal protection measures, and periodic assessment of TB infection control is essential in healthcare facilities and congregate settings. Although we did not perform periodic assessments, data from this study can be used as baseline information.

This study had some limitations. First, TB skin tests were not performed, therefore, we could not compare the correlation of TB skin tests and IGRA. However, according to previous studies, IGRA is the better tool for diagnosis of LTBI. Second, although this was the large-scale study consisting of >1000 subjects and involving all types of hospital HCWs, the population was not representative of HCWs in South Korea because this was a single-center study.

In conclusion, this study reported a 16% prevalence rate of LTBI in HCWs by IGRA. LTBI among HCWs in Korea, although prevalent, might not exceed the background level of the general population. Because contact with active TB patients has been identified as a risk factor for LTBI, more effective TB infection control measures are essential in healthcare facilities and congregate settings.
